# The Sex Differences in Regulating Unpleasant Emotion by Expressive Suppression: Extraversion Matters

**DOI:** 10.3389/fpsyg.2016.01011

**Published:** 2016-07-07

**Authors:** Ayan Cai, Yixue Lou, Quanshan Long, Jiajin Yuan

**Affiliations:** ^1^The Laboratory for Affect Cognition and Regulation, Key Laboratory of Cognition and Personality of Ministry of Education, Faculty of Psychology, Southwest UniversityChongqing, China; ^2^School of Psychology, Southwest UniversityChongqing, China

**Keywords:** sex difference, extraversion, expressive suppression, event-related potentials, late positive potentials

## Abstract

Males are known for more suppression of emotional displays than females. However, when the emotion regulation effect of expressive suppression is greater in males, and how this sex difference varies with emotion display-related personality (e.g., extraversion), are undetermined. Event-related potentials were recorded while male and female participants different in extraversion were required to attend to or suppress emotional expression to negative pictures. Sex and extraversion did not modulate self-reported emotional experience. However, late positive potential (LPP) amplitudes showed an extraversion-moderated sex difference in the 2000–3000 ms and the 3000–4000 ms time epochs. LPP amplitudes were decreased during suppression versus viewing conditions in ambivert males, while this effect was absent in ambivert females. However, the LPP amplitudes of extraverts were similar for suppression and viewing conditions, irrespective of sex and timing. Regardless of early, middle, or late time windows, LPP amplitudes were positively related to self-reported emotion. These results suggest a male advantage for using expressive suppression for emotion regulation in non-extraverted, ambivert individuals.

## Introduction

Expressive suppression is a widely used emotion regulation strategy, and this strategy has been proved particularly effective in regulating emotional consequences in East Asian societies (Butler et al., 2007; Yuan et al., 2014a,b). The suppression of inappropriate emotion-expressive behaviors, especially unpleasant ones, plays an important role in humans’ social adaptation and the maintenance of relational harmony (Kitayama et al., 2000; Mesquita, 2001; Murata et al., 2013). As we known, males are less emotionally expressive in humans’ daily communication and engage more emotion-expressive suppression than females, supported by a number of empirical studies (Buck et al., 1974; Kring et al., 1994; Barrett et al., 1998; Kring and Gordon, 1998; Hess et al., 2000; Parkins, 2012; Chaplin and Aldao, 2013). For example, females cry more often than males (Lombardo et al., 1983; Ross and Mirowsky, 1984). Matud (2004) has observed that females have more chronic and daily stress than males, which is closely associated with sex differences in stress coping: Females used more emotional and avoidance coping styles, whereas males adopted more rational coping and emotional-expressive suppression (Matud, 2004). Using the Emotion Regulation Questionnaire (ERQ), Gross and John (2003) found that males scored significantly higher than females in suppression scales when investigating individual differences in emotion regulation. This finding was confirmed by subsequent researchers using ERQ (Haga et al., 2009). Chen et al. (2005) examined the construct of ambivalence over emotion expression in Chinese culture, and found that males are more likely to suppress emotions than females. It is noteworthy that what these research measured is the frequency of using suppression strategy, rather than how effectively the suppression strategy regulates emotional reaction. Despite abundant research showing more frequent suppression of emotional expressions in males than in females, little evidence has shown that males are better than females in dampening negative emotions by expressive suppression. A recent study in our lab, using an experiment to explore sex differences in emotion regulation, has found that males did outperform females in regulating negative emotion by expressive suppression (Cai et al., 2016). It is noteworthy that the effect of sex observed in this study is based on general population, and it may not apply to specific individuals. Particularly, the sex differences in expressive suppression may be moderated by personality trait like extraversion.

Prior studies have also indicated that the tendency of emotional expressiveness is moderated by personality traits like extraversion (Riggio and Riggio, 2002; 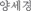 and OhSoosung, 2009). Extraversion is an emotion-related personality trait characterized by the tendency to experience positive emotions, activity, and sociability (Lischetzke and Eid, 2006; Tamir, 2009; Yuan et al., 2009). Along the personality dimension of extraversion, people scoring high are considered as extraverts who are described as being outgoing, talkative, impulsive and uninhibited, with many social contacts and being frequently involved in group activities (Costa and McCrae, 1992; Ashton et al., 2002). By contrast, those scoring low are described as quiet, retiring, introspective, not socially active (Costa and McCrae, 1992; Krentzman et al., 2012) and reluctant to communicate through facial displays (Riggio and Riggio, 2002), as is typical of introverts. It has been indicated that higher extraversion is associated with greater excitability, increased emotional expressivity and better communication of non-verbal emotional information (Buck et al., 1972; Riggio and Riggio, 2002; Chen et al., 2005). Consistent with these findings, there is recent evidence showing that high extraversion is associated with increased difficulty in the suppression of emotional displays (Peña-Gómez et al., 2011). However, no study to date has tested whether emotion display-related personality traits, like extraversion, influence the sex differences in regulation of unpleasant emotion by expressive suppression. Based on the evidence described above, we hypothesized that expressive suppression is most likely ineffective for regulating negative emotion in extraverts, irrespective of sex, and that the sex difference in regulating negative emotion by expressive suppression just applies to a less extraverted population.

To directly test whether the effect of gender in regulating unpleasant emotions by expressive suppression varies as a function of extraversion, the current study used the event-related potential (ERP) technique, by classifying subjects into different groups according to sex and extraversion. Numerous researchers have shown that late positive potential (LPP), a posterior-parietal positive slow ERP (Hajcak and Nieuwenhuis, 2006; Foti and Hajcak, 2008) that reaches its largest amplitudes at 500–700 ms post-stimulus and lasts for several 100 ms, was more pronounced for emotionally salient than for neutral stimuli (Cuthbert et al., 2000; Schupp et al., 2000, 2004). Moreover, LPP amplitude has been accepted as an ideal index for the intensity of emotional experience (Cuthbert et al., 2000; Amrhein et al., 2004; Olofsson et al., 2008). The LPP amplitudes decrease with the reduction of emotional experience during emotion regulation (Hajcak and Nieuwenhuis, 2006; Moser et al., 2006, 2010; Krompinger et al., 2008; Thiruchselvam et al., 2011). Thus, the LPP in brain potentials was chosen as a direct index in the current study to study the temporal dynamics of emotion arousal during expressive suppression and its modulations by sex and extraversion. We hypothesized that LPP amplitudes of ambivert males are significantly smaller during expressive suppression compared to viewing conditions, and this amplitude reduction would be less prominent in ambivert females. By contrast, this emotion regulation effect is probably absent in extraverts, irrespective of sex, based on the studies mentioned above.

## Materials and Methods

### Subjects

As paid volunteers, 68 right-handed undergraduate students with no history of major psychiatric or neurological disorders participated in this experiment. All of the subjects completed the NEO Five-Factor Inventory (NEO-FFI, Chinese version; internal consistency coefficient = 0.878; Wang et al., 2010), a five-point (from -2 to 2), 240-item questionnaire that is widely used in personality assessments (Canli et al., 2002; Amin et al., 2004). The four experimental samples were determined by subjects’ sex and scores in the extraversion subscale (48 items; internal consistency coefficient = 0.88; Wang et al., 2010) of the NEO-FFI.

**FIGURE 1:**
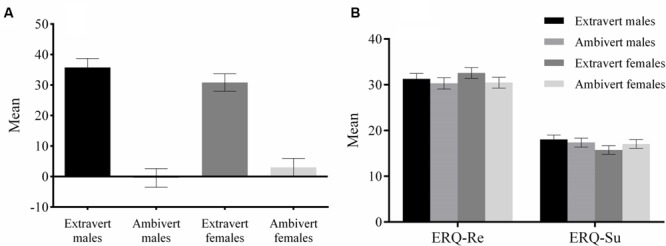
**Means of the scores on the Extraversion subscale of the NEO-FFI **(A)** and ERQ **(B)** for the four groups.** The error bar represents standard error.

Participants whose extraversion scores were above the 50th percentile were categorized as extraverts, while the rest were categorized as ambiverts. We labeled subjects whose extraversion scores were below the 50th percentile as ambiverts rather than introverts, because their extraversion scores were centered around zero, the neutral point along the extraversion-introversion continuum (see **Figure 1**). The 4-group subjects, including 17 extravert males (aged 18–29, mean 21.4; extraversion score: *M* ±*SE*: 35.76 ± 2.96), 16 ambivert males (aged 18–24, mean 21.06; extraversion score: -0.44 ± 3.05), 18 extravert females (aged 17–25, mean 20.89; extraversion score: 30.83 ± 2.88) and 17 ambivert females (aged 17–23, mean 20.53; extraversion score: 3.0 ± 2.96), were similar in the habitual use of cognitive reappraisal [*F*(3,64) = 0.77, *p* = 0.51, $\eta_{p}^{2}$ = 0.04] and expressive suppression [*F*(3,64) = 1.06, *p* = 0.37, $\eta_{p}^{2}$ = 0.05] in the Emotion Regulation Questionnaire (ERQ). *T*-test showed no significant differences in extraversion scores between extravert males and extravert females [*t*(33) = 1.06; *p* = 0.30], nor between the ambivert males and ambivert females [*t*(31) = -0.95; *p* = 0.35]. Additionally, the extraversion scores were significantly different between extravert males and ambivert males [*t*(31) = 7.30, *p* < 0.001], and between extravert females and ambivert females [*t*(33) = 8.26, *p* < 0.001; see **Figure 1**]. We measured the neuroticism subscale of the NEO Personality Inventory to ensure that all subjects were emotionally stable, since neuroticism assesses affective adjustment and emotional instability (Piedmont, 2000). Indeed, neuroticism assesses six facets, including anxiety, angry hostility, depression, self-consciousness, impulsiveness, and vulnerability. The significant below-threshold (0) score in anxiety [*t*(67) = 6.31, *p* < 0.001] and depression [*t*(67) = 8.30, *p* < 0.01] subscales of neuroticism assessment, suggested that our subjects were emotionally stable and free of depression and anxiety. The averaged depression (or anxiety) scores were -7.35 (or -5.12) for extravert males, -2.63 (or -0.38) for ambivert males, -5.39 (or -3.17) for extravert females, and -3.29 (-3.24) for ambivert females. The participants of both samples were right handed and had normal or corrected to normal vision. The study was approved by the local Review Board for Human Participant Research, and each participant signed an informed consent form before the experiment. The study was conducted following the ethical principles of the Helsinki Declaration regarding human experimentation (World Medical Organization, 1996).

### Stimuli and Procedures

**FIGURE 2:**
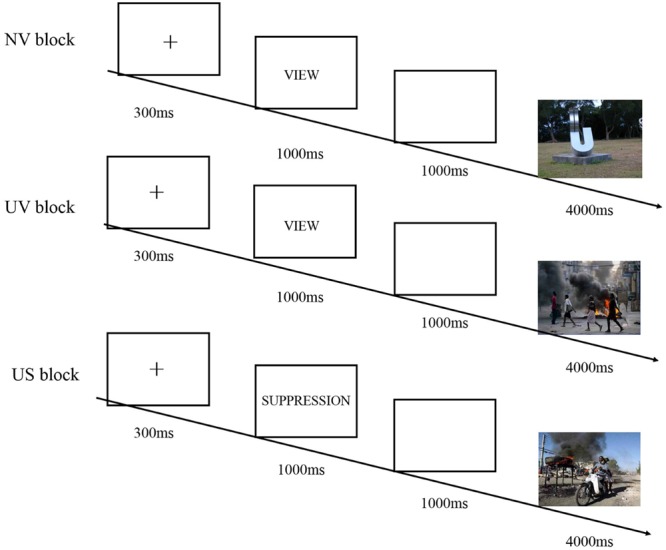
**Schematic illustration of the experimental procedure**.

The present study used a block-design picture viewing task (see **Figure 2**). The task consisted of three blocks, and each block included 40 picture stimuli that were taken from the International Affective Picture System (IAPS) and its Chinese adapted Version (Chinese Affective Picture System, CAPS). The picture stimuli were neutral, emotionally irrelevant in the first block, as a non-emotional baseline for computing emotion effect in later conditions (Neutral-View, NV). The last two blocks required subjects to either view 40 unpleasant pictures without using any emotion regulation strategies (Unpleasant-View, UV) or view pictures while regulating unpleasant emotion by expressive suppression (Unpleasant-Suppression, US). The order of the UV and US blocks was counterbalanced across subjects. Unpleasant pictures were composed of the scenes of frightening animals, human attack and body mutilations while neutral pictures depicted the scenes of neutral animals and human activities.

In order to avoid emotional habituation or sensitization when a single set of pictures are repeatedly presented, the current study randomly selected two different sets of unpleasant pictures for the UV and US. Also, there was evidence showing a cultural bias when IAPS was applied to Chinese subjects (Bai et al., 2005). In order to control these influences and attribute differences in dependent variables solely to emotion regulation, we conducted a separate procedure checking whether the two sets of pictures were similar in emotional parameters for Chinese people (Yuan et al., 2014b). For this purpose, four trained judges (two males) blind to research purposes were invited to rate the valence (9-point scale, from 1: extremely unpleasant to 9: extremely pleasant) and arousal (9-point scale, from 1: very calm to 9: very excited) of the pictures. The four judges were highly consistent in assessing the emotionality of the pictures. The inter-rater reliability (Kendall’s coefficient of concordance) was 0.83 [χ^2^(3) = 9.9; *p* < 0.05] for both valence and arousal dimensions. Therefore, we averaged the rating data across the four judges for each picture, and then conducted a one-way ANOVA for the valence and arousal of pictures with experimental condition as a predictor. The condition effect for arousal was highly significant [*F*(2,117) = 318.85, *p <* 0.001]. The pairwise comparisons showed significantly higher arousal values for UV (*M* = 7.20) and US (6.97) conditions than the NV (3.03) condition (*p*s < 0.001). In addition, the arousal values were not significantly different during the UV and US conditions (*p* = 0.21 for UV-US). Similarly, the condition effect for valence was also significant [*F*(2,117) = 312.74, *p <* 0.001]. The pairwise comparisons showed significantly lower valence values for UV (1.73) and US (1.83) conditions in comparison with the NV (4.71) condition (*p*s < 0.001). In addition, the valence values were not significantly different during the UV and US conditions (*p* = 0.43). Thus, the pictures used for UV and US conditions were valid in inducing unpleasant emotions, and the emotion attributes of the pictures were kept similar across the two unpleasant conditions.

Subjects were seated in a quiet room approximately 150 cm from the computer screen with the horizontal and vertical visual angles below 6°. Prior to each block, subjects were instructed on how to do the task and were presented with eight trials for practice, where the eight pictures were emotionally similar to the pictures used in the experiment. In block NV and UV, each trial was initiated by a small black fixation cross on the white computer screen for 300 ms. The offset of the cross was followed by a 1000 ms presentation of a word “view,” reminding subjects of the task in this block. Then, a 1000 ms blank screen was presented, followed by the onset of pictures for 4000 ms. *Subjects were instructed to pay close attention and react normally to each stimulus, and experience any feelings it elicited naturally* (Ohira et al., 2006). Between blocks; 2 min of rest, which was the maximal time used by another 10 subjects to rest in a pilot study, were used for subjects to recover their mood to the baseline level.

In the US block, the stimulus stream was the same as that of the NV and the UV, except that the word changed into “suppression,” reminding subjects to use expressive suppression to regulate unpleasant emotion. Participants were trained in the suppression strategy during practice trials. *Suppression instructions trained participants to intentionally suppress the expression of emotion responses to pictures, by keeping their facial expressions unchanged so that someone watching their face would be unable to detect what was being experienced subjectively* (Goldin et al., 2008). At the end of each block, subjects were required to rate their mood state by a self-report 7-point scale (1: neutral, non-emotional to 7: extremely unpleasant). Also, they were asked to rate how successfully they suppressed emotion-expressive behaviors or attended to the pictures by a 7-point scale (1: not successful at all; 7: completely successful). At the end of the experiment, subjects rated the pleasantness of every picture (1: extremely unpleasant; 5: neutral, non-emotional; 9: extremely pleasant).

### ERP Recording and Analysis

The EEG was recorded from 64 scalp sites using tin electrodes mounted in an elastic cap (Brain Products, Munich, Germany), with the reference electrodes on the left and right mastoids (average mastoid reference; Luck, 2005), and the ground electrode on the medial of the frontal aspect. The Vertical electrooculograms (EOGs) were recorded below the right eye, and the horizontal EOGs were recorded on the right side of the right eye. The EEG and EOG were amplified using a DC ∼100 Hz band-pass and were continuously sampled at 500 Hz/channel. All inter-electrode impedance was maintained below 5 kΩ. The averaging of ERPs was computed off-line. Eye movement artifacts (blinks and eye movements) were corrected oﬄine and a 24-Hz low-pass filter was used. Trials with EOG artifacts (mean EOG voltage exceeding ±100 μV) and those contaminated with artifacts due to amplifier clipping of peak-to-peak deflection that exceeded ±100 μV were excluded from averaging. Rejected trials were rare. There was an average of 38.49 trials for NV, 38.83 trials for UV, and 38.90 trials for US conditions obtained for ERP averaging. EEG in each block was averaged separately. The ERP waveforms were time-locked to the onset of stimuli and the averaged epoch for ERPs was 4500 ms including a 500 ms pre-stimulus baseline. According to the literature, LPP is a positive slow wave of the ERP with a centroparietal midline maximum scalp distribution (Cuthbert et al., 2000; Schupp et al., 2000; Hajcak and Nieuwenhuis, 2006). We measured average amplitudes for LPP amplitudes at midline central and centroparietal regions (six sites: C1, Cz, C2, CP1, CPz, and CP2), and segmented 500–4000 ms into three consecutive time windows, 500–2000, 2000–3000, and 3000–4000 ms, separately representing early, middle, and late windows of LPP, according to the results of Principal Component Analysis (PCA, see Supplementary Material) (Smith et al., 2003).

We took two steps for ERP analysis: (1) A repeated-measure ANOVA of LPP amplitudes in each time window was conducted to examine whether unpleasant pictures validly induced unpleasant emotional arousal (with stimulus type [NV, UV] as a within-subjects factor while sex [males, females] and extraversion [extravert, ambivert] as between-subjects factors). (2) A repeated-measures ANOVA was conducted to examine the emotional regulation effect (with regulation strategy [UV, US] as within-subjects factor, sex [males, females] and extraversion [extravert, ambivert] as between-subjects factors). Since the present study focused on the moderation of extraversion on emotional regulation of males and females to unpleasant stimuli, we focused the statistical analysis on the three-way interaction involving regulation strategy, sex and extraversion. The degrees of freedom of the *F*-ratio were corrected according to the Greenhouse–Geisser method for any violations of sphericity, and the Bonferroni–Holm method was used to adjust the *p*-value during *post hoc* pairwise comparisons if significant main or interaction effects were detected. The effect sizes were shown as partial eta squared ($\eta_{p}^{2}$).

## Results

### Manipulation Check

The analysis of the instruction confirmation data (responses to the question “how successfully did you attend to the pictures, or suppress your expression of emotion?”) showed that subjects successfully attended to the pictures during NV (*M* = 5.32) and UV conditions (6.16), and successfully suppressed their emotion expression during the US condition (6.35). The scores for each condition were significantly higher than the midpoint of the rating scale (i.e., 4) [*t*(67) = 6.68–25.93, *p*_s_ <0.001]. The instruction confirmation was not significantly different between UV and US conditions [*t*(67) = -1.72, *p* > 0.09].

### The Mood Assessment

#### Emotional Arousal Effect

**FIGURE 3:**
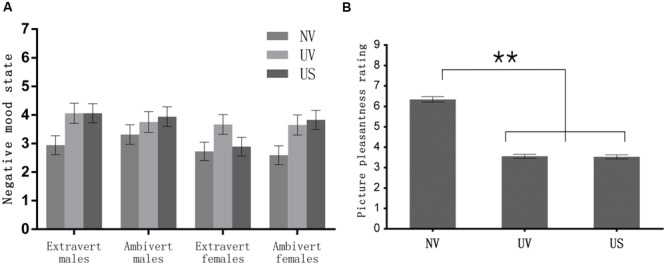
**The results of mood rating for each condition **(A)**, and Means of the picture pleasantness assessment in each condition **(B)**.** ^∗∗^*p* < 0.001.

The repeated-measures ANOVA of mood rating scores, with stimulus type (NV, UV) as within-subjects factor, and sex and extraversion as the between-subjects factors, showed no other significant main or interaction effects (all *p*s > 0.19), except for a significant main effect of stimulus type [*F*(1,64) = 18.33, *p* < 0.001, $\eta_{p}^{2}$ = 0.23]. Follow-up contrast showed that the negative affect was significantly greater when watching negative pictures (3.78) than neutral pictures (2.89), irrespective of sex or extraversion (see **Figure 3**).

#### Emotion Regulation Effect

We conducted a 2(Sex) × 2(Extraversion) × 2(regulation strategy: UV, US) repeated-measure ANOVA. The results showed no significant main effects or interaction effects (all *p*s > 0.06).

### The ERP Analysis

#### LPP: Early Window (500–2000 ms)

##### Emotional arousal effect

The results showed a significant main effect of stimulus type [*F*(1,64) = 35.99, *p* < 0.001, $\eta_{p}^{2}$ = 0.36]. The emotional arousal effect was due to the larger LPP amplitudes for negative (2.05 μV) versus neutral (-0.11 μV) pictures. The interactions between factors were not significant (all *p*s > 0.06).

##### Emotional regulation effect

Neither main effects nor interactions between factors reached statistical significance (all *p*s > 0.08).

#### LPP: Middle Window (2000–3000 ms)

##### Emotional arousal effect

The results only showed a significant main effect of stimulus type [*F*(1,64) = 21.76, *p* < 0.001, $\eta_{p}^{2}$ = 0.25], with larger amplitudes elicited for negative (1.51 μV) versus neutral (-0.22 μV) pictures.

##### Emotion regulation effect

**FIGURE 4:**
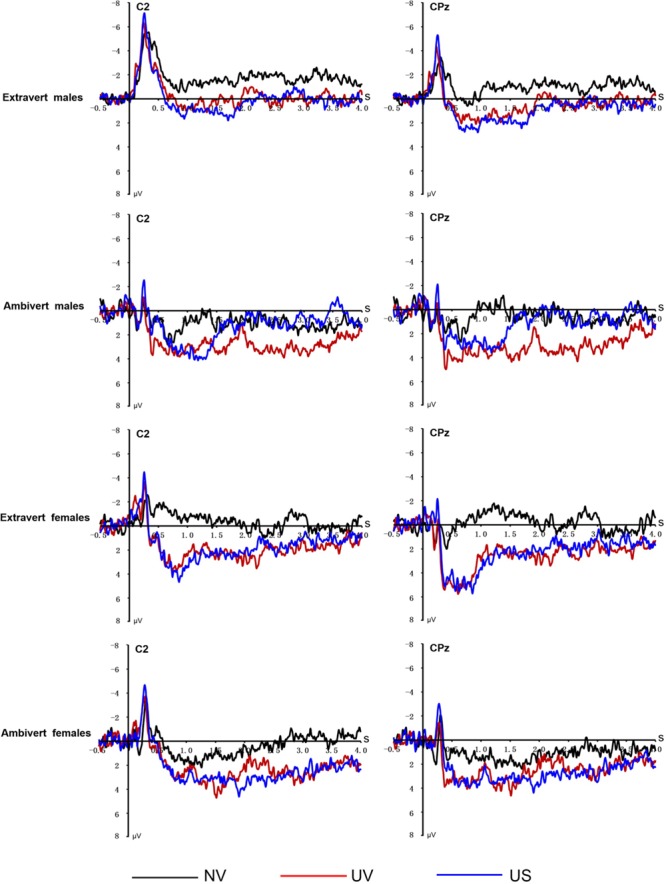
**Averaged ERPs elicited by NV, UV, US conditions at C2 and CPz electrode sites in each group**.

**FIGURE 5:**
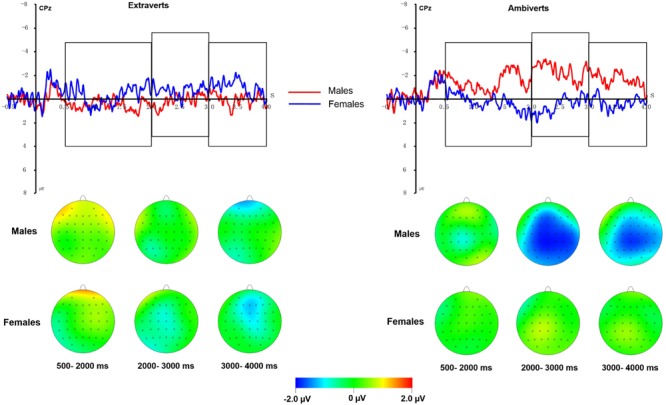
**(Top) US-UV difference waveform at CPz for males (red) and females (blue) in the extravert and ambivert samples. (Bottom)** Topographical maps of the voltage amplitudes of US-UV difference waves for males and females in extraverts and ambiverts from 500 to 4000 ms post stimulus.

There was a significant three-way interaction of regulation strategy, sex and extraversion [*F*(1,64) = 8.63, *p* = 0.005, $\eta_{p}^{2}$ = 0.20]. We tested the interaction of sex and regulation strategies in extravert and ambivert subjects, respectively. In the ambivert group, there was a significant sex by strategy interaction [*F*(1,31) = 11.60, *p* = 0.002, $\eta_{p}^{2}$ = 0.27], which was analyzed by examining the emotion regulation effect in ambivert males and ambivert females, separately. The regulation effect was significant in ambivert males [*F*(1,15) = 8.27, *p* = 0.012, $\eta_{p}^{2}$ = 0.36] but not in ambivert females [*F*(1,16) = 3.02, *p* = 0.10, $\eta_{p}^{2}$ = 0.16; see **Figures 4** and **5**]. Ambivert males displayed smaller amplitudes during US condition (0.46 μV) than during UV condition (2.35 μV). By contrast, the sex by strategy interaction [*F*(1,33) = 0.98, *p* = 0.33, $\eta_{p}^{2}$ = 0.03], and the main effect of sex or strategy (all *p*s > 0.31), were all non-significant in extraverts. This implies that there was no significant emotion regulation effect in extraverted groups, irrespective of sex.

To assess the timing of these results, a 5 (segment: 2000–2200, 2200–2400, 2400–2600, 2600–2800, and 2800–3000 ms) × 2 (regulation strategy) × 2 (sex) × 2 (extraversion) repeated-measures ANOVA was performed. Neither the main effect of a segment [*F*(4,154) = 2.10, *p* = 0.12, $\eta_{p}^{2}$ = 0.03] nor the interactions involving a segment and other factors (all *p*s > 0.09) reached significance. This suggests that the above findings are reliable, as neither overall LPP amplitudes nor the group-specific emotion regulation effect varied across time segments in the 2000–3000 ms.

#### LPP: Late Window (3000–4000 ms)

##### Emotional arousal effect

The results showed a significant main effect of stimulus type [*F*(1,64) = 13.88, *p* < 0.001, $\eta_{p}^{2}$ = 0.18]. The unpleasant pictures (1.23 μV) elicited larger LPP amplitude relative to neutral pictures (-0.19 μV). The interactions between factors were not significant (all *p*s > 0.57).

##### Emotional regulation effect

We observed a significant three-way interaction of regulation strategy, sex and extraversion [*F*(1,64) = 6.31, *p* = 0.015, $\eta_{p}^{2}$ = 0.09]. We then analyzed the sex by regulation interaction in extraverts and ambiverts, respectively. The sex by regulation interaction [*F*(1,31) = 6.32, *p* = 0.017, $\eta_{p}^{2}$ = 0.17] was significant in ambiverts. The subsequent analysis showed a significant emotion regulation effect in ambivert males [*F*(1,15) = 4.60, *p* = 0.049, $\eta_{p}^{2}$ = 0.24], with smaller amplitudes during the US condition (-0.21μV) than during the UV condition (1.70μV), whereas the effect was not significant in ambivert females [*F*(1,16) = 1.51, *p* = 0.24, $\eta_{p}^{2}$ = 0.09; see **Figures 4** and **5**]. However, there was neither a significant main effect of regulation (*p* > 0.21), nor a significant sex by regulation interaction in the extravert group [*F*(1,33) = 1.02, *p* = 0.32, $\eta_{p}^{2}$ = 0.03], suggesting that expressive suppression produced no significant emotion regulation effect in extraverts, irrespective of sex.

Also, neither the main effect of a segment [*F*(4,147) = 0.84, *p* = 0.45, $\eta_{p}^{2}$ = 0.01] nor the interactions involving a segment (all *p*s > 0.11) reached significance in the analysis of the timing effect, suggesting that neither overall amplitudes nor the group-specific regulation effect varied across time in the 3000–4000 ms.

### Emotion Assessment

The repeated-measures ANOVA of picture pleasantness ratings, with conditions as repeated factor, sex and extraversion as the between-subjects factors, showed no other significant main or interaction effects (*p*s > 0.18), except for a significant main effect of condition [*F*(1,67) = 215.57, *p* <0.001, $\eta_{p}^{2}$ = 0.77]. Subjects rated UV (*M* ±*SE*: 3.56 ± 0.10; *p <* 0.001) and US (3.53 ± 0.10; *p* < 0.001, see **Figure 3**) pictures as more unpleasant than NV (6.35 ± 0.14) pictures. In addition, the rating scores for the UV [*t*(67) = -14.97, *p* < 0.001] and US [*t*(67) = -14.64, *p* < 0.001] pictures were both significantly lower than the midpoint of the rating scale (i.e., 5), while the ratings were not significantly different between UV and US conditions [*t*(67) = 0.94, *p* = 0.35]. Thus, the pictures used for UV and US conditions were valid in inducing unpleasant emotion, and the pictures’ unpleasant strength was similar across UV and US conditions.

### Correlation Analyses

**FIGURE 6:**
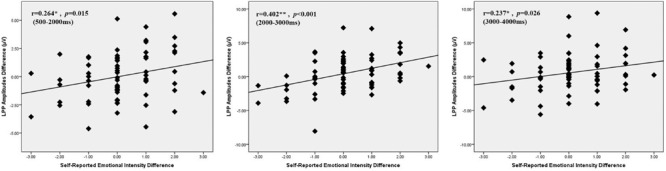
**Change in LPP amplitude (UV-US) plotted as a function of change in self-reported emotional intensity (view minus suppression) in 500–2000, 2000–3000, 3000–4000 ms.** Each dot represents an individual participant.

To verify whether LPP amplitudes during the US condition reflect subjective emotion intensity, a Pearson correlation was computed between the LPP amplitude differences and subjective emotion differences during UV relative to US conditions in the LPP time window. We found that the reduction of subjective emotional intensity increased significantly with the LPP amplitude reduction in each of the three LPP windows (see **Figure 6**).

## Discussion

Negative pictures elicited greater unpleasant feelings, and greater positive amplitudes across each time window of LPP component in comparison with neutral pictures, suggesting that our selection of negative pictures was successful. Though all the samples showed no significant reduction in emotional experiences after suppressing unpleasant emotions, ambivert males, rather than ambivert females, were associated with significantly smaller LPP amplitudes during expressive suppression relative to viewing conditions in the 2000–4000 ms at midline, central, and centroparietal regions. But LPP amplitudes of extraverts were similar for suppression and viewing conditions in all time windows, irrespective of sex.

On the one hand, the current study observed that expressive suppression produced a better regulation of unpleasant emotional reactions in ambivert men than in ambivert women, as reflected by the LPP amplitude differences between UV and US conditions. Ambiverts, a group of non-extraverted and non-introverted persons located in the median of the bell-shaped distribution of extraversion (McCrae and Costa, 2003; Yuan et al., 2012; Grant, 2013), are more representative of the general population when compared with typical extraverts or introverts. Thus, the above findings may have implications for the general population in that expressive suppression of negative emotion produces better, more adaptive emotional physiological consequences in males than in females. LPP has been accepted to serve as an electrophysiological marker of emotional arousal evoked by salient pictures (Hajcak and Nieuwenhuis, 2006; Moser et al., 2006; Krompinger et al., 2008). Consistent with the evidence, the current study observed a significant positive correlation between LPP amplitudes and self-reported emotion intensity during viewing versus suppression conditions. This further suggests that LPP amplitude is most likely a physiological reflection of subjective emotional arousal, and that LPP amplitude reduction in males versus females represents sex differences in the reduction of emotional reaction.

Prior studies have indicated a systematic sex difference in emotional expression. Women reported more intense emotional expression and feelings, and a greater tendency to seek emotional experiences than men (Allen and Hamsher, 1974; Allen and Haccoun, 1976; Balswick and Avertt, 1977; Larsen and Diener, 1987; Hampson et al., 2006) and exhibited greater facial EMG activity during the viewing of emotion-inducing slides (Grossman and Wood, 1993), in comparison to men. Kring and Gordon (1998) required subjects to view film clips including happy, sad and fear types. They observed that, compared with men, women were more facially expressive, though they did not differ from men in reports of experienced emotion. Grossman and Wood (1993) pointed out that this sex difference may be due to the sex role diversification between males and females, and this explanation was later confirmed by the study of Kring and Gordon (1998). Haga et al. (2009) explained that emotion-expressive suppression is central to the norms of masculinity and is consistent with the cultural expectations for masculine gender-role; that is, men are expected not to show as much emotion as women and are therefore strongly encouraged to suppress their feelings (Broverman et al., 1972; Roseman, 1984; Eccles et al., 1990; Brooks, 1998). Males may unconsciously follow the norms of masculinity to suppress their unpleasant emotions in daily life, consequently leading to a greater skill at regulating unpleasant emotion by suppressing emotional expression. However, the current study did not directly assess gender roles. Thus, we need to be cautious with this gender role explanation. Whether the gender role mediates the male advantage at regulating unpleasant emotion with expressive suppression needs direct examination in future studies.

On the other hand, the results confirmed our hypothesis that this pattern of sex differences disappeared in extraverts. At the higher side of the continuum of extraversion, none of the extravert males and extravert females effectively decreased unpleasant emotions by expressive suppression. Amin et al. (2004) found that participants scoring high in extraversion exhibited significantly faster RTs in a dot-probe attention task, when the probe was placed behind the neutral rather than behind the negative stimulus locations when a negative/neural composite picture was used. This suggests that extraverts tend to shift their attention away from a negative stimulus, and they may be inclined to adopt other strategies such as distraction to regulate unpleasant emotion. There was abundant evidence showing that higher extraversion is linked with more emotional expression and less emotion-expressive suppression (Carver and Scheier, 2000; Gross and John, 2003; Chen et al., 2005). For instance, Gross and John (2003) observed that trait extraversion is negatively associated with habitual suppression of emotional expression, with higher extraversion predicting less emotion-expressive suppression. This negative correlation was later replicated by Chen et al. (2005), who further demonstrated that extraverts are less ambivalent over emotional expression, that is, more consistent in internal expressive intention and overt expressive behaviors, in comparison with those lower in trait extraversion. Furthermore, Peña-Gómez et al. (2011) recently reported that the improvement of emotional inhibition decreased as a function of increasing extraversion, when subjects received anodal transcranial direct current stimulation of the prefrontal inhibitory network. In this regard, it is probably the increased tendency for emotional expression, which characterizes people high in extraversion, that has contributed to the extraverts’ absence of emotion regulation effect during expressive suppression in the current study, irrespective of sex.

One may question that the ERQ assessment showed similar self-reported suppression across the four samples, which may contradict abundant evidence of sex differences in suppression. However, in real life settings, the self-reported suppression is not equal to the actual use of suppression, as many studies indicate that emotion regulation strategies may work unconsciously, in the absence of overt instructions, particularly as a result of training (e.g., Tran et al., 2011; Heeren et al., 2012). For instance, after the training of attention focus on non-emotional or positive stimuli, socially anxious participants showed significantly reduced experiential anxiety and decreased physiological activations when watching negative facial expressions as compared to those without training, though there was no explicit request of emotion regulation (Heeren et al., 2012). This effect is not merely prominent in adults but is also observable in children (MacLeod and Holmes, 2012). Similarly, there is abundant evidence that subjects receiving positive interpretation training tend to automatically interpret novel situations as positive, showing reduced negative emotional consequences during stress induction, despite no explicit instruction of positive interpretation (Wilson et al., 2006; Tran et al., 2011). It has been suggested that the culturally shaped or personality- determined coping style works rather automatically, and the practice of habitual coping is more an unconscious process than a controlled deliberate process (Mauss et al., 2007). Thus, due to social or cultural training, ambivert males down-regulated unpleasant emotional reaction to a greater extent by expressive suppression than females, though they are not necessarily conscious of more suppression. On the other hand, prior studies consistently indicate that the more an adaptive strategy is used, the higher the emotion regulation effect is. For instance, shifting attention from negative to positive stimuli is linked with extraversion-related happiness (Amin et al., 2004), higher reappraisal is linked with decreased negative affect (Gross and John, 2003), and greater suppression is linked with reduced negative emotion in Asian cultures (Butler et al., 2007). Thus, the fact that we observed this pattern of sex difference after controlling for self-reported suppression in itself strengthens the validity of the current finding. This suggests that extraversion-moderated sex difference exists reliably, irrespective of whether or not ambivert males are conscious of more habitual suppression than females.

## Author Contributions

JY designed experiments; AC, YL, and QL carried out experiments; AC analyzed experimental results. AC and JY wrote the manuscript.

## Conflict of Interest Statement

The authors declare that the research was conducted in the absence of any commercial or financial relationships that could be construed as a potential conflict of interest.
