# Supplementary material for: The Sex Differences in Regulating Unpleasant Emotion by Expressive Suppression: Extraversion Matters
Source: Front Psychol. 2016 Jul 7;7:1011. doi: 10.3389/fpsyg.2016.01011 (PMC4935688; doi:10.3389/fpsyg.2016.01011)
Supplement: Supplementary file 1 [file Data_Sheet_1.DOCX]

**Supplementary material**

Principal Component Analysis (PCA)

LPP is a slow positive potential lasting for several seconds. It is necessary to check different phases of LPP and separate them accordingly. For this purpose, a Principal Component Analysis (PCA) was conducted on the LPP amplitudes (Smith, Cacioppo, Larsen, & Chartrand, 2003; J. Yuan et al., 2012). LPP is a positive component starting from 400-500ms and lasting for several seconds, and this component was largest at the posterior-parietal scalp region (Foti & Hajcak, 2008; Hajcak & Nieuwenhuis, 2006). The details are shown as follows.

For PCA, a set of averaged ERP waveforms are statistically examined for covariations in amplitude across time points. From patterns of covariation across time points, scalp sites, and participants, the PCA decomposes a set of average wave-forms into a small number of voltage×time functions (called components) with each component representing a portion of the overall variance in the set input waveforms. Each component consists of one covariance (called a component loading) for each time point in the ERP waveforms, with the loading indicating the extent to which that component has an influence on that time point. Therefore, higher loadings indicate time points where a component is strongly active, whereas small loadings indicate time points where components are relatively inactive. In addition to the component loadings, PCA also generates a set of component scores. These scores indicate the extent to which a component is present in a given waveform (Smith, Cacioppo, Larsen, & Chartrand, 2003). As mentioned previously, PCA analyzes the patterns of covariation between time points in the average waveforms in order to consolidate the total variance of the waveforms down into the activity of a small number of underlying components. To do this, difference waves as the subject averages were entered into the PCA as a voltage × time function. To ensure that the PCA’s components would be consistent with the underlying neural functioning, the output of the PCA was subjected to a varimax rotation and five components were extracted between 0 and 4000ms. The component scores were then saved for each component by subject by electrode by average combination and analyzed using the GLM module of SPSS. The results of the PCA were as follows:

Graphing the component loadings versus time yields a depiction of the time-points over which the components exhibit influence. The five components between 0 and 4000ms extracted by the PCA are depicted. The numbering of the components represents the temporal order in which each component is strongly active. The percentage written in red color represents the proportion of variance accounted for by each component.

In the time interval from 0 to 4000ms, there were five principal components labeled component 1, 2, 3, 4 and 5. If these components are indeed LPP, it should possess the characteristic latency, scalp distribution, and polarity of the LPP. That is, Component should begin 400-500ms after stimulus onset and last for several seconds. In addition, Component should be largest at posterior-parietal sites. Furthermore, the component should be positive going at the sites where the component amplitude was largest.

For the LPP, to address the second criterion, we analyzed the mean component scores for the component 3 (500-2000ms) across all the scalp sites. We found that LPP was largest over the centroparetial electrode site (Fz: M=-0.11，S.E.=0.55；FCz: M=-0.14，S.E.=0.65；Cz: M=0.88，S.E.=0.61；CPz: M=3.65，S.E.=0.57；Pz: M=0.97，S.E.=0.58). Finally, in order to examine the polarity of the component at the centroparetial site, the component score at this site must be multiplied by the component loading at its peak value. Because both of these values are positive, the component is positive going at CPz. Based on these three criteria, the component 3 should be LPP.

We analyzed the mean component scores for the component 5 (2000-3000ms) across all the scalp sites. We found that LPP was largest over the centroparetial electrode site (Fz: M=1.22，S.E.=0.62；FCz: M=2.03，S.E.=0.69；Cz: M=1.47，S.E.=0.54；CPz: M=2.44，S.E.=0.50；Pz: M=1.79，S.E.=0.65). Finally, in order to examine the polarity of the component at the centroparetial site, the component score at this site must be multiplied by the component loading at its peak value. Because both of these values are positive, the component is positive going at CPz. Based on these three criteria, the component 5 should be LPP.

For the LPP, to address the second criterion, we analyzed the mean component scores for the component 1 (3000-4000ms) across all the scalp sites. We found that LPP was largest over the centroparetial electrode site (Fz: M=0.84，S.E.=0.51；FCz: M=0.71，S.E.=0.73；Cz: M=0.54，S.E.=0.52；CPz: M=1.89，S.E.=0.43；Pz: M=-0.98，S.E.=0.78). Finally, in order to examine the polarity of the component at the centroparetial site, the component score at this site must be multiplied by the component loading at its peak value. Because both of these values are positive, the component is positive going at CPz. Based on these three criteria, the component 1 should be LPP.


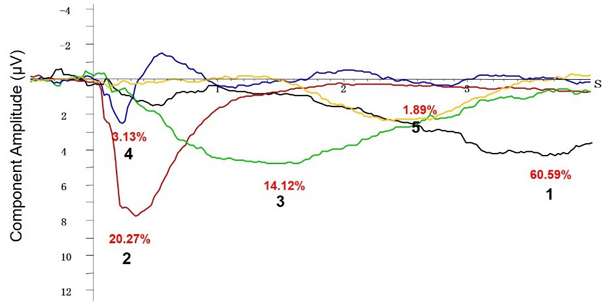


Component amplitude as a function of time. Percentage of variance explained by component is also indicated (%)
